# Book Review: *Point of Care Ultrasound in Critical Care* by Luke Flower and Pradeep Madhivathanan

**DOI:** 10.1177/17511437231213527

**Published:** 2023-11-20

**Authors:** Peter G Brindley

**Affiliations:** University of Alberta, Edmonton, AB, Canada

Because so much medical learning is now delivered by Dr. Google, the textbook faces an uncertain, potentially perilous, future. Regardless, this Intensive Care Doctor still enjoys flicking pages; especially when the book in question is a one-stop primer covering one of my blind spots. If, like me, you are yet to fully master your ultrasound skills, then ‘Point of Care Ultrasound in Critical Care’ edited by Flower and Madhivathanan is worth your precious time and cash. Kudos to these editors, and to each of contributing ultrasound gurus: they have ably summarized what ‘luddites’, like me, need.

Full disclosure, this – cough cough – late-career intensivist was not an ultrasound early adopter, but nor was I ever a ‘refuse-nik’. Instead, I was that ‘weekend warrior’ who took ultrasound courses, but did not fully immerse. This means that I still need to regularly refresh, and this text serves that purpose. If your ultrasound skills can be described with the same words that a Francophone colleague used to summarize my French language skills – namely ‘enthusiastic but modest’ – then you are, like me, this book’s target audience. I can happily report that I am now, if not ultrasound fluent, then at least more able to get around.

The authors and publishers deserve further kudos because this 28-chapter book is Goldilocks-long (340 pages), and affordably priced (approximately 40 GBP, 50 USD). Opening chapters cover the basics of ultrasound physics, and probes, and how to operate machines. After all, without good pictures our clinical decisions will be impaired (‘garbage-in garbage-out’). The book then quickly pivots into an organ by organ review, with sequential focus on cardiac, lung, abdominal, vascular, neurologic and airway. It finishes with concise chapters on situational ultrasound; namely the assessment of shock, fluid status, trauma, cardiac arrest and weakness. I previously relied upon You Tube for my ultrasound instruction, because it is obvious where the probe was placed, orientated and moved. Fortunately, in a similar fashion, this text provides accompanying video clips.

Any downsides? Well yes, but none that I blame on the authors. For example, just like mastering a language, few will master ultrasound with books alone. Instead, you need to be literally ‘hands on’: with a probe, patient, and problem. Moreover, while ultrasound has taken the old school guess work (albeit, educated guesswork) out of clinical decision-making, there is an ever-present danger. In other words, sonographic idolatry could replace taking a proper history, performing a focused physical exam or knowing your physiology inside and out. Ultrasound is now core curriculum, so we ALL need to get on board. This does not mean, however, that every part of the body must be scanned before we proceed.

Ultrasound is powerful but no panacea. Moreover, to my knowledge, no ultrasound text has addressed when less is more. After all, these (otherwise terrific) devices can potentially occupy ‘resuscitative real estate’, obscure sight lines and distract us from immediate concerns (they are called ‘vitals’ for a reason). Indiscriminate ultrasound might even create ‘analysis paralysis’, or annoyance when we restate the obvious (‘Doctor, your patient with known Heart Failure, has a poorly functioning heart’).

None of this, hopefully respectful, critique should take away from celebrating all that critical care ultrasound offers. The sonographic high priests are unlikely to need this book but are likely to ‘echo’ my delight that everyone is learning. The challenge will be to maintain those skills and to grow ultrasound as an academic discipline, not just a training priority. Ultimately, this technology, and book, will only be as good as the clinician that uses it. To quote a certain arachnid superhero, ‘with great power comes great responsibility’. Regardless, critical care ultrasound is already something to ‘Marvel^TM^’ at.

